# Enhancement of absorption in a CH_3_NH_3_PbI_3_-based photonic crystal in the presence of the monolayer MoS_2_

**DOI:** 10.1038/s41598-023-33261-5

**Published:** 2023-04-12

**Authors:** Farhad Sattari, Soghra Mirershadi

**Affiliations:** 1 Department of Physics, Faculty of Sciences, University of Mohaghegh Ardabili, Ardabil, P.O. Box 179, Iran; 2 Nanoscience and Nanotechnology Research Group, University of Mohaghegh Ardabili, Ardabil, Iran; 3 Department of Engineering Sciences, Faculty of Advanced Technologies, University of Mohaghegh Ardabili, Namin, Iran

**Keywords:** Materials science, Optics and photonics, Physics

## Abstract

Using the transfer matrix approach, we investigate theoretically the absorbance, transmittance, and reflectance through one-dimensional CH_3_NH_3_PbI_3_ perovskite-based photonic crystal at room temperature. In our proposed structure, a monolayer MoS_2_ film is embedded between two CH_3_NH_3_PbI_3_ layers. We found that, the presence of monolayer MoS_2_ film increases the absorbance in longer wavelengths $$(\lambda > 600\begin{array}{*{20}c} \, \, {\rm nm} \\ \end{array} ).$$ With increasing the number of periods, absorbance increases in most wavelengths of the incident light. It was shown that, by controlling the number of periods, the absorbance coefficient can be tuned according to the wavelength and angle of incident light. Furthermore, for incident light with longer wavelength, the absorbance, transmittance as well as reflectance versus thickness of the perovskite layer have an oscillatory behavior, and with increasing the number of periods this oscillatory behavior becomes more obvious and prominent. For the incident light in the infrared region, by increasing the number of periods the absorbance as opposed to the transmittance increases for different incidence angles. While, the reflectance coefficient first shows oscillatory behavior by increasing the number of periods, then with a further increase in the number of periods it reaches a constant value. The proposed structure can be useful for optoelectronic and optical devices. Such as improving the efficiency of solar cells based on the hybrid inorganic–organic perovskites and infrared sensor system.

## Introduction

In recent years, hybrid inorganic–organic perovskites CH_3_NH_3_PbX_3_ (X = Cl^−^, Br^−^, I^−^) are a promising potential for photovoltaic and optoelectronic applications, for examples lasers^1^, solar cells^2^, photodetectors^3^ and light-emitting diodes^4^ due to their unique properties, such as suitable and adjustable energy gap^5^, high photoluminescence quantum yield^6^, high mobility of charge carriers^7^ and high exciton binding energy at room temperature^8^. Among these perovskites, CH_3_NH_3_PbI_3_ (methylammonium lead triiodide perovskite) has more applications in optoelectronic and photovoltaic devices, due to its properties such as energy band gap and sharp absorption edge^9^. For example, the highest power conversion among thin film solar cells has been reported for CH_3_NH_3_PbI_3_^10^. Also, after the discovery of graphene in 2004^11^ as the first two-dimensional (2D) material, other 2D materials such as phosphorene^12^, borophene^13^, silicene^14^, hexagonal boron nitride (*h*-BN)^15^, gyroidal graphene^16^ and transition metal dichalcogenides (TMDs)^17^ were successfully synthesized and characterized. These 2D materials have attracted much attention, due to their interesting mechanical, electronic and optical properties. One of the most important members of TMDs is monolayer molybdenum disulfide (MoS_2_)^18^. Due to its properties such as direct and adjustable energy bandgap^19^, thermal stability^20^ and complex refractive index^21^, it can be widely used in optical devices such as, solar cells^22^, phototransistors^23^ and light emitters^24^. On the other hand, the photonic crystal is a periodic optical structure, which is made of layers with different and periodic refractive indices^25^. Because the lattice constant in photonic crystals can be easily engineered by the thickness of the layers. Therefore, they are the important structures for controlling the propagation of light and sensing applications^26,27^. Recently, the propagation of electromagnetic waves through photonic crystals, especially photonic crystals based on the 2D materials, has been widely investigated both theoretically and experimentally by researchers and significant results have been obtained^28–50^. The optical absorbance of graphene on the one-dimensional photonic crystal has been investigated theoretically by Liu et al.^28^ The authors showed that, when graphene is placed on the photonic crystal, its absorbance coefficient increases greatly. Also, they found that, the absorbance coefficient can be easily controlled by tuning the angle of incident light and the distance between photonic crystal and graphene. Kuang et al.^31^ experimentally proposed a photonic crystal on crystalline silicon. It is found that, when the thickness of crystalline silicon is $$500\begin{array}{*{20}c} \, {\upmu m} \\ \end{array}$$, for a wide range of incident light wavelengths $$(\lambda = 400 - 1000\begin{array}{*{20}c}\, \, {\rm nm} \\ \end{array} ),$$ the transmittance and absorbance coefficients are approximately equal to zero and one, respectively. Tunable ultrafast absorption by BaTiO_3_‑based photonic crystal was investigated in Ref.^40^. It is demonstrated that, this novel photonic crystal can be useful in design and fabrication of nonlinear optical filter, optical limiters and optical switches. Ansari et al*.*^45^ investigated the electromagnetic wave propagating in the monolayer transition-metal dichalcogenides-based photonic crystal by transfer matrix method, and observed that in this photonic crystal, the absorbance coefficient is more than 75% for all incident light angles with a wavelength of 400 to 675 nm. Further, they obtained that, in WS_2_-based photonic crystal the absorbance coefficient for incident light with a wavelength of 619 nm reaches 100%. Also, inserting of the defect inside the photonic crystal (such as SiO_2_) can significantly increase the transmittance coefficient, which can have various applications, for example narrowband transmittance filter^51^. Our aim in this paper was to investigate the propagation of electromagnetic waves in a CH_3_NH_3_PbI_3_ perovskite-based photonic crystal, which has not investigated to date to the best of our knowledge. In our proposed photonic crystal, a monolayer MoS_2_ film is embedded between two CH_3_NH_3_PbI_3_ layers. In this paper we demonstrated that, the absorbance coefficient, increases by increasing the number of periods. The amount of absorbance can be controlled by tuning the angle of incident light. Moreover, the absorbance, transmittance and reflectance coefficients have an oscillatory behavior versus thickness of the perovskite layer, and with increasing the number of periods this oscillatory behavior becomes more obvious. Remarkably, the absorption of CH_3_NH_3_PbI_3_-based photonic crystal increases in the presence of the monolayer MoS_2_, so this structure can be useful for optoelectronic and optical devices. Such as improving the efficiency of solar cells based on the hybrid inorganic–organic perovskites and luminescent solar concentrator^52^. The rest of this article is as follows: the proposed structure, which is a one-dimensional photonic crystal based on CH_3_NH_3_PbI_3_ perovskite, is given in “Model and methods”. Also, in this section, the absorbance, transmittance and reflectance coefficients in the proposed structure are calculated based on the transfer matrix approach. “Results and discussion” is devoted to discuss the results of the numerical calculations. “Conclusion” summarizes the main findings of this study.

## Model and methods

**Figure 1:**
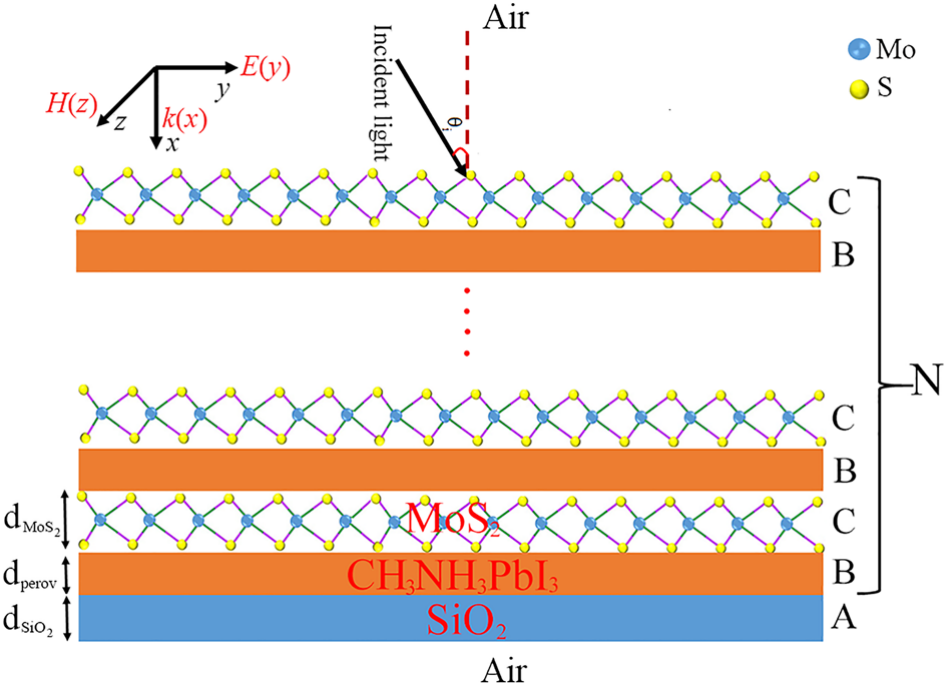
Schematic of the CH_3_NH_3_PbI_3_ perovskite-based photonic crystal, in which a monolayer MoS_2_ film is embedded between two CH_3_NH_3_PbI_3_ layers.

In this work, we consider the CH_3_NH_3_PbI_3_ perovskite-based photonic crystal, in which a monolayer MoS_2_ film is embedded between two CH_3_NH_3_PbI_3_ layers. The proposed structure i.e., Air/(*CB*)^*N*^*A*/Air is showed in Fig. 1, where *N* is the number of periods. *A*, *B* and *C* are SiO_2_, CH_3_NH_3_PbI_3_ and MoS_2_ layers, respectively. The thickness of layers SiO_2_, MoS_2_ and CH_3_NH_3_PbI_3_ are considered as $$d_{{SiO_{2} }} ,$$$$d_{{MoS_{2} }}$$ and $$d_{Perovskite}$$, respectively.

In order to obtain the absorbance, transmittance, and reflectance coefficients of the electromagnetic wave incident on the proposed photonic crystal, we must know the refractive indices of the different layers used in the photonic crystal. The refractive index of SiO_2_ $(n_{{SiO_{2} }} )$ can be calculated by the following relations^53^:

1 $$ \begin{gathered} \hfill \\ n_{{SiO_{2} }} (\lambda ) = \sqrt {A_{S} + \frac{{B_{S} \lambda^{2} }}{{\lambda^{2} - C_{S}^{2} }} + \frac{{D_{S} \lambda^{2} }}{{\lambda^{2} - E_{S}^{2} }}} . \hfill \\ \end{gathered} $$

Here,$$\begin{array}{*{20}c} {A_{S} = 1.2860441} \\ \end{array} ,$$$$B_{s} = 1.07044083,C_{s} = 0.10029,E_{s} = 10$$ and $$D_{s} = 1.10202242$$^53^. $$\lambda$$ is the incident light wavelength in micrometers. Further, the air refractive index is set to 1 (top and bottom sides of the photonic crystal). Due to the complex nature of the refractive index of monolayer MoS_2_, Lorentz–Drude–Gaussian method can be used to obtain this refractive index^54^. In this method, the electric permittivity of the monolayer MoS_2_ can be obtained by the following relationship:

2 $$ \varepsilon_{{MoS_{2} }}^{{}} = \varepsilon_{{rMoS_{2} }}^{{}} + i\varepsilon_{{iMoS_{2} }}^{{}} = \varepsilon_{{MoS_{2} }}^{LD} + \varepsilon_{{MoS_{2} }}^{G} . $$

where, $\varepsilon_{{MoS_{2} }}^{LD}$ and $\varepsilon_{{MoS_{2} }}^{G}$ are the parts of the Lorentz-Drude and Gaussian, respectively and are given by the following relations:

3 $$ \varepsilon_{{MoS_{2} }}^{LD} = \varepsilon_{\infty } + \sum\limits_{j = 0}^{5} {\frac{{s_{j} \omega_{p}^{2} }}{{\omega_{j}^{2} - \omega_{{}}^{2} - i\Gamma_{j} \omega }}} , $$

4 $$ \begin{gathered} \varepsilon_{{rMoS_{2} }}^{G} = 1 - \frac{{2\varepsilon_{I} }}{\sqrt \pi }DF(\frac{\hbar \omega - E}{{\sqrt 2 \sigma }}), \hfill \\ \varepsilon_{{iMoS_{2} }}^{G} = \varepsilon_{I} \exp ( - \frac{{(\hbar \omega - E)^{2} }}{{2\sigma^{2} }}). \hfill \\ \end{gathered} $$

Here, $$\sigma = 0.3089$$ eV, $$E = 2.7723$$ eV and $$\varepsilon_{I} = 23.224$$. *DF* is the Dawson function. $$\omega_{p} = (28.3/2\pi )$$ meV represent the plasma frequency for monolayer MoS_2_ and $$\varepsilon_{\infty } = 4.44$$ displays the DC permittivity. $$s_{j} ,$$$$\omega_{j}$$ and $$\Gamma_{j} ,$$ are the *j*-th strength of the oscillator, resonance frequency and damping coefficients, respectively. The values of these quantities for the first six resonances are given in Ref^55^. Also, $$\varepsilon_{{rMoS_{2} }}^{G}$$ and $$\varepsilon_{{iMoS_{2} }}^{G}$$ are the real and imaginary components of the Gaussian part of the dielectric permittivity of the monolayer MoS_2_. The imaginary $$(\varepsilon_{{iMoS_{2} }}^{{}} )$$ and real $$(\varepsilon_{{rMoS_{2} }}^{{}} )$$ parts of the dielectric permittivity of monolayer MoS_2_ can be calculated after obtaining the $$\varepsilon_{{MoS_{2} }}^{LD}$$ and $$\varepsilon_{{iMoS_{2} }}^{G}$$, then the refractive index of the monolayer MoS_2_ $$(n^{\prime}_{{MoS_{2} }} )$$ can be easily obtained from the following equation:

5 $$ \begin{gathered} n_{{MoS_{2} }} (\omega ) = \sqrt {\frac{1}{2}(\varepsilon_{{rMoS_{2} }} + \sqrt {\varepsilon_{{_{{rMoS_{2} }} }}^{2} + \varepsilon_{{_{{iMoS_{2} }} }}^{2} } )} , \hfill \\ K_{{MoS_{2} }} (\omega ) = \sqrt {\frac{1}{2}( - \varepsilon_{{rMoS_{2} }} + \sqrt {\varepsilon_{{_{{rMoS_{2} }} }}^{2} + \varepsilon_{{_{{iMoS_{2} }} }}^{2} } )} , \hfill \\ n^{\prime}_{{MoS_{2} }} (\omega ) = n_{{MoS_{2} }} (\omega ) + iK_{{MoS_{2} }} (\omega ), \hfill \\ \end{gathered} $$

where, $n_{{MoS_{2} }}$ and $K_{{MoS_{2} }}$ are the real and imaginary part of the refractive index of the monolayer MoS_2,_ respectively. Also, in order to calculate the complex refractive index of the CH_3_NH_3_PbI_3_ layer $(n^{\prime}_{perovskite} = n_{perovskite} + iK_{perovskite} ),$ the imaginary and real part of the dielectric permittivity of this material is needed. Here, we have used the experimentally obtained values for dielectric permittivity of the CH_3_NH_3_PbI_3_ film at room temperature in Ref^56^. According to Fig. 1, we assume that the electromagnetic wave impinges upon the proposed photonic crystal with an angle of $\theta_{i}$ to the *x*-axis, thus in the* j*th layer with refractive index $n_{j}$, the electric field component of TE mode can be written in the following form:

6 $$ \begin{gathered} E_{j} (y) = A_{j} e^{{ik_{j} x}} + B_{j} e^{{ - ik_{j} x}} , \hfill \\ k_{j} = \sqrt {k_{0}^{2} n_{j}^{2} - \beta^{2} } = k_{0} n_{j} \cos \theta_{j} , \hfill \\ k_{0} = \frac{2\pi }{\lambda }, \hfill \\ \beta = k_{0} n_{j} \sin \theta_{j} , \hfill \\ \theta_{j} = \cos^{ - 1} \left( {\sqrt {1 - \frac{{n_{0}^{2} \sin \theta_{i}^{2} }}{{n_{j}^{2} }}} } \right). \hfill \\ \end{gathered} $$

where, $n_{0} = 1$ is the air refractive index and $\theta_{j}$ displays the incident angle in the* j*th layer. Also, in the *j*th layer, the magnetic field tangential component is related to the electric field by the following equation:

7 $$ \begin{gathered} H_{j} (z) = - \frac{i}{\omega }\frac{{\partial E_{j} (y)}}{\partial x} = \alpha_{j} (A_{j} e^{{ik_{j} x}} - B_{j} e^{{ - ik_{j} x}} ), \hfill \\ \alpha_{j} = \frac{{k_{0} n_{j} \cos \theta_{j} }}{\omega }. \hfill \\ \end{gathered} $$

Using the continuity of the magnetic and electric fields at the boundary of the layers and applying the transfer matrix method^57^, the transmittance, reflectance and absorbance coefficients can be obtained in the investigated system. Thus, by applying the boundary conditions for the first layer with air and the second layer, the following relationships are obtained:

8 $$ \left( {\begin{array}{*{20}c} 1 \\ r \\ \end{array} } \right) = \left( {\begin{array}{*{20}c} 1 & 1 \\ {\alpha_{0} } & { - \alpha_{0} } \\ \end{array} } \right)^{ - 1} \left( {\begin{array}{*{20}c} 1 & 1 \\ {\alpha_{1} } & { - \alpha_{1} } \\ \end{array} } \right)\left( {\begin{array}{*{20}c} {A_{1} } \\ {B_{1} } \\ \end{array} } \right), $$

9 $$ \left( {\begin{array}{*{20}c} {A_{1} } \\ {B_{1} } \\ \end{array} } \right) = - \frac{1}{{2\alpha_{1} }}\left( {\begin{array}{*{20}c} { - \alpha_{1} e^{{ - ik_{1} d_{{MoS_{2} }} }} } & { - e^{{ - ik_{1} d_{{MoS_{2} }} }} } \\ { - \alpha_{1} e^{{ik_{1} d_{{MoS_{2} }} }} } & {e^{{ik_{1} d_{{MoS_{2} }} }} } \\ \end{array} } \right)\left( {\begin{array}{*{20}c} {e^{{ik_{2} d_{{MoS_{2} }} }} } & {e^{{ - ik_{2} d_{{MoS_{2} }} }} } \\ {\alpha_{2} e^{{ik_{2} d_{{MoS_{2} }} }} } & { - \alpha_{2} e^{{ik_{2} d_{{MoS_{2} }} }} } \\ \end{array} } \right)\left( {\begin{array}{*{20}c} {A_{2} } \\ {B_{2} } \\ \end{array} } \right), $$

By replacing Eq. (9) in Eq. (8), we can write:

10 $$ \begin{gathered} \left( {\begin{array}{*{20}c} 1 \\ r \\ \end{array} } \right) = \left( {\begin{array}{*{20}c} 1 & 1 \\ {\alpha_{0} } & { - \alpha_{0} } \\ \end{array} } \right)^{ - 1} m_{1} \left( {\begin{array}{*{20}c} {e^{{ik_{2} d_{{MoS_{2} }} }} } & {e^{{ - ik_{2} d_{{MoS_{2} }} }} } \\ {\alpha_{2} e^{{ik_{2} d_{{MoS_{2} }} }} } & { - \alpha_{2} e^{{ik_{2} d_{{MoS_{2} }} }} } \\ \end{array} } \right)\left( {\begin{array}{*{20}c} {A_{2} } \\ {B_{2} } \\ \end{array} } \right) \hfill \\ m_{1} = \left( {\begin{array}{*{20}c} {\cos \delta_{1} } & { - \frac{i}{{\alpha_{1} }}\sin \delta_{1} } \\ { - \alpha_{1} \sin \delta_{1} } & {\cos \delta_{1} } \\ \end{array} } \right),\begin{array}{*{20}c} {\begin{array}{*{20}c} {} & {\delta_{1} } \\ \end{array} } \\ \end{array} = k_{1} d_{{MoS_{2} }} \cos \theta_{1} ,\begin{array}{*{20}c} {k_{1} = k_{0} n^{\prime}_{{MoS_{2} }} \cos \theta_{1} .} & {} \\ \end{array} \hfill \\ \hfill \\ \end{gathered} $$

Also, by applying the boundary conditions for the second layer and using Eq. (10), the following relationship can be written:

11 $$ \begin{gathered} \left( {\begin{array}{*{20}c} 1 \\ r \\ \end{array} } \right) = \left( {\begin{array}{*{20}c} 1 & 1 \\ {\alpha_{0} } & { - \alpha_{0} } \\ \end{array} } \right)^{ - 1} m_{1} m_{2} \left( {\begin{array}{*{20}c} {e^{{ik_{3} l_{3} }} } & {e^{{ - ik_{3} l_{3} }} } \\ {\alpha_{3} e^{{ik_{3} l_{3} }} } & { - \alpha_{3} e^{{ik_{3} l_{3} }} } \\ \end{array} } \right)\left( {\begin{array}{*{20}c} {A_{3} } \\ {B_{3} } \\ \end{array} } \right) \hfill \\ m_{2} = \left( {\begin{array}{*{20}c} {\cos \delta_{2} } & { - \frac{i}{{\alpha_{2} }}\sin \delta_{2} } \\ { - \alpha_{2} \sin \delta_{2} } & {\cos \delta_{2} } \\ \end{array} } \right),\begin{array}{*{20}c} {\begin{array}{*{20}c} {} & {\delta_{2} } \\ \end{array} } \\ \end{array} = k_{2} d_{perovskite} \cos \theta_{2} ,\begin{array}{*{20}c} {k_{2} = k_{0} n_{perovskite} \cos \theta_{2} ,} & {} \\ \end{array} \hfill \\ l_{3} = d_{{MoS_{2} }} + d_{perovskite} . \hfill \\ \end{gathered} $$

Similarly, by using the boundary conditions for the third layer and according to Eq. (11), the following equation can be obtained:

12 $$ \begin{gathered} \left( {\begin{array}{*{20}c} 1 \\ r \\ \end{array} } \right) = \left( {\begin{array}{*{20}c} 1 & 1 \\ {\alpha_{0} } & { - \alpha_{0} } \\ \end{array} } \right)^{ - 1} m_{1} m_{2} m_{3} \left( {\begin{array}{*{20}c} {e^{{ik_{4} l_{4} }} } & {e^{{ - ik_{4} l_{4} }} } \\ {\alpha_{4} e^{{ik_{4} l_{4} }} } & { - \alpha_{4} e^{{ik_{4} l_{4} }} } \\ \end{array} } \right)\left( {\begin{array}{*{20}c} {A_{4} } \\ {B_{4} } \\ \end{array} } \right) \hfill \\ m_{3} = \left( {\begin{array}{*{20}c} {\cos \delta_{3} } & { - \frac{i}{{\alpha_{3} }}\sin \delta_{3} } \\ { - \alpha_{3} \sin \delta_{3} } & {\cos \delta_{3} } \\ \end{array} } \right),\delta_{3} = k_{3} d_{{SiO_{2} }} \cos \theta_{3} ,k_{3} = k_{0} n_{{SiO_{2} }} \cos \theta_{3} , \hfill \\ l_{4} = d_{{MoS_{2} }} + d_{perovskite} + d_{{SiO_{2} }} . \hfill \\ \theta_{3} = \cos^{ - 1} \left( {\sqrt {1 - \frac{{n_{0}^{2} \sin^{2} \theta_{i} }}{{n_{{SiO_{2} }}^{2} }}} } \right). \hfill \\ \end{gathered} $$

Due to, the complex refractive index of the CH_3_NH_3_PbI_3_ and monolayer MoS_2_, $\theta_{1}$ and $\theta_{2}$ are given by^58^:

13 $$ \theta_{1} = \sin^{ - 1} (\frac{{n_{0} \sin \theta_{i} }}{{\sqrt {\frac{1}{2}\sqrt {(n_{{MoS_{2} }}^{2} - K_{{MoS_{2} }}^{2} - n_{0}^{2} \sin^{2} \theta_{0} )^{2} + 4n_{{MoS_{2} }}^{2} K_{{MoS_{2} }}^{2} } + (n_{{MoS_{2} }}^{2} - K_{{MoS_{2} }}^{2} ) + n_{0}^{2} \sin^{2} \theta_{i} } }}). $$

14 $$ \theta_{2} = \sin^{ - 1} (\frac{{n_{0} \sin \theta_{i} }}{{\sqrt {\frac{1}{2}\sqrt {(n_{perovskite}^{2} - K_{perovskite}^{2} - n_{0}^{2} \sin^{2} \theta_{0} )^{2} + 4n_{perovskite}^{2} K_{perovskite}^{2} } + (n_{perovskite}^{2} - K_{perovskite}^{2} ) + n_{0}^{2} \sin^{2} \theta_{i} } }}), $$

For the proposed photonic crystal with length *L* and the number of periods *N*, by applying the boundary conditions and using the transfer matrix method, the following relations can be written:

15 $$ \begin{gathered} \left( {\begin{array}{*{20}c} 1 \\ r \\ \end{array} } \right) = \left( {\begin{array}{*{20}c} 1 & 1 \\ {\alpha_{0} } & { - \alpha_{0} } \\ \end{array} } \right)^{ - 1} M\left( {\begin{array}{*{20}c} {e^{{ik_{0} L}} } & 0 \\ {\alpha_{0} e^{{ik_{0} L}} } & 0 \\ \end{array} } \right)\left( {\begin{array}{*{20}c} t \\ 0 \\ \end{array} } \right), \hfill \\ M =^{{}} (m_{1} m_{2} )^{N} (m_{3} ), \hfill \\ \end{gathered} $$

16 $$ t = \frac{{2\alpha_{0} e^{{ - ik_{0} L}} }}{{(M_{11} + M_{12} \alpha_{0} )\alpha_{0} + (M_{21} + M_{22} \alpha_{0} )}}, $$

17 $$ \begin{gathered} r = \frac{{M_{11} \alpha_{0} - M_{21} + (M_{12} \alpha_{0} - M_{22} )\alpha_{0} }}{{(M_{11} + M_{12} \alpha_{0} )\alpha_{0} + (M_{21} + M_{22} )\alpha_{0} }}, \hfill \\ T = tt^{*} = \left| t \right|^{2} ,\begin{array}{*{20}c} {} & {R = rr^{*} = \left| r \right|^{2} ,} \\ \end{array} \begin{array}{*{20}c} {} & {A = 1 - T - R.} \\ \end{array} \hfill \\ \end{gathered} $$

Here, *A*,* R* and *T* are the absorbance, reflectance and transmittance coefficients of a CH_3_NH_3_PbI_3_ perovskite-based photonic crystal.

## Results and discussion

**Figure 2:**
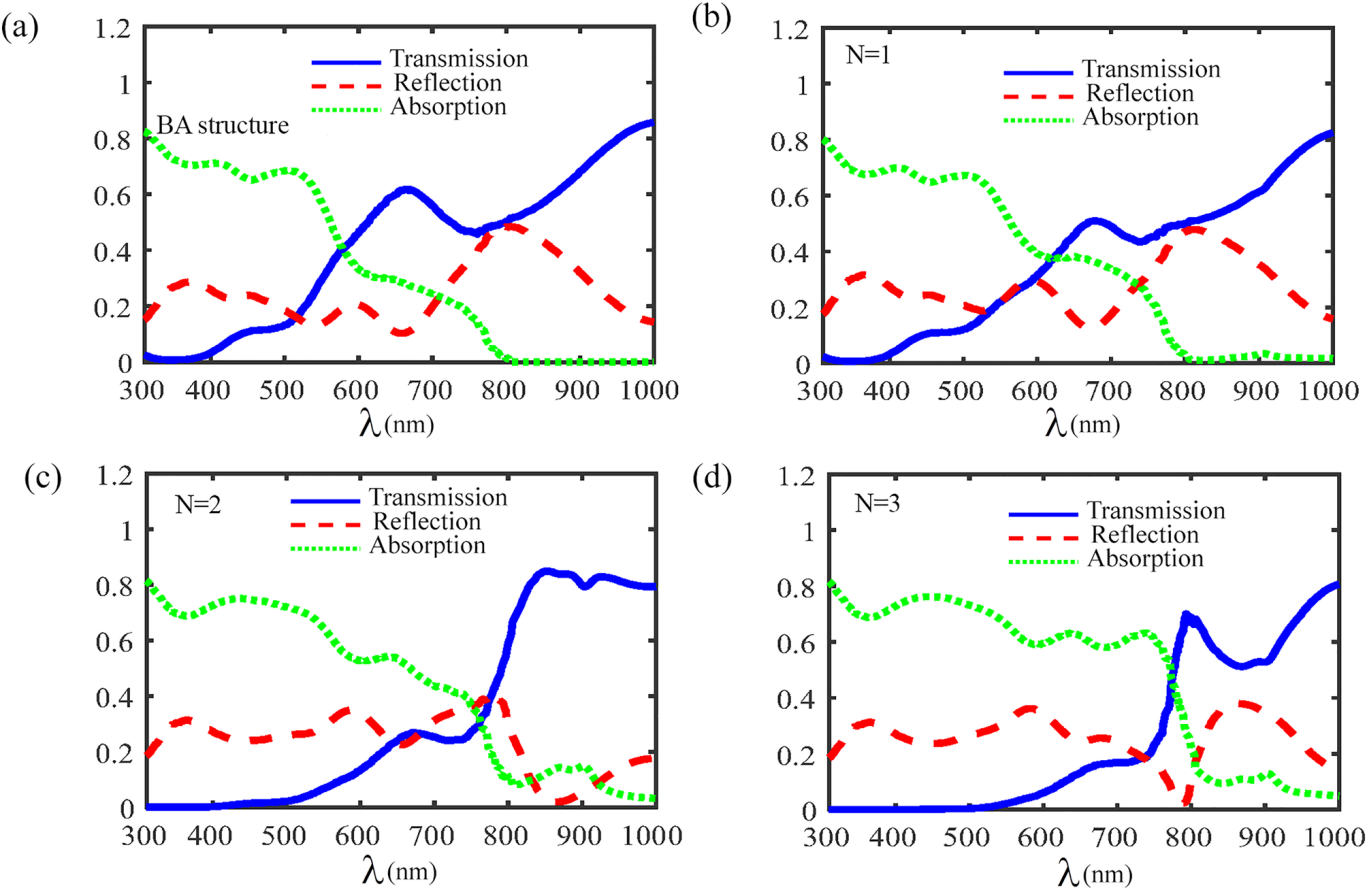
The absorbance, transmittance and reflectance coefficients versus light wavelength for the normal incident angle $\theta_{i} = 0^{o}$, (**a**) for *BA* structure, (**b**) *N* = 1, (**c**) *N* = 2 and (**d**) *N* = 3.

In the following, we focus on the absorbance, reflectance and transmittance coefficients in a CH_3_NH_3_PbI_3_ perovskite-based photonic crystal based on the formulas derived in the previous section. In the present calculation we fix the $d_{{SiO_{2} }} = 500$ nm, $d_{{MoS_{2} }} = 0.65$ nm and $d_{perovskite} = 100$ nm. First, the transmittance, reflectance and absorbance coefficients as a function of incident light wavelength for the normal incident angle $\theta_{i} = 0^{o}$, and for various the number of periods are plotted in Fig. 2. As it is clear from the figure, for *BA* structure the absorbance coefficient of the structure is zero for longer wavelengths $$(\lambda > 800\begin{array}{*{20}c} \, \, {\rm nm} \\ \end{array} ).$$ Because the imaginary part of dielectric constant for CH_3_NH_3_PbI_3_ is zero in these wavelengths^56^. While, for *N* = 1 the photonic crystal has a weak absorbance around 900 nm, due to the presence of the monolayer MoS_2_. Considering that the CH_3_NH_3_PbI_3_ layer and monolayer MoS_2_ alone have very weak absorption in longer wavelengths^52,59,60^. However, due to the optical localization in CH_3_NH_3_PbI_3_-based photonic crystal in the presence of the monolayer MoS_2,_ this structure enhances the light absorption at longer wavelengths. This phenomenon will be more evident with the increase in the number of periods. Also, as the number of periods increases, the absorbance coefficient increases, especially at longer wavelengths. In addition, for *BA* structure or* N* = 1, the transmittance coefficient of the photonic crystal is zero over a small wavelengths range $$(300\begin{array}{*{20}c} \, {\rm nm} \\ \end{array} < \lambda < 400\begin{array}{*{20}c} \, {\rm nm} \\ \end{array} ),$$ and this range increases with increasing the number of periods.

**Figure 3:**
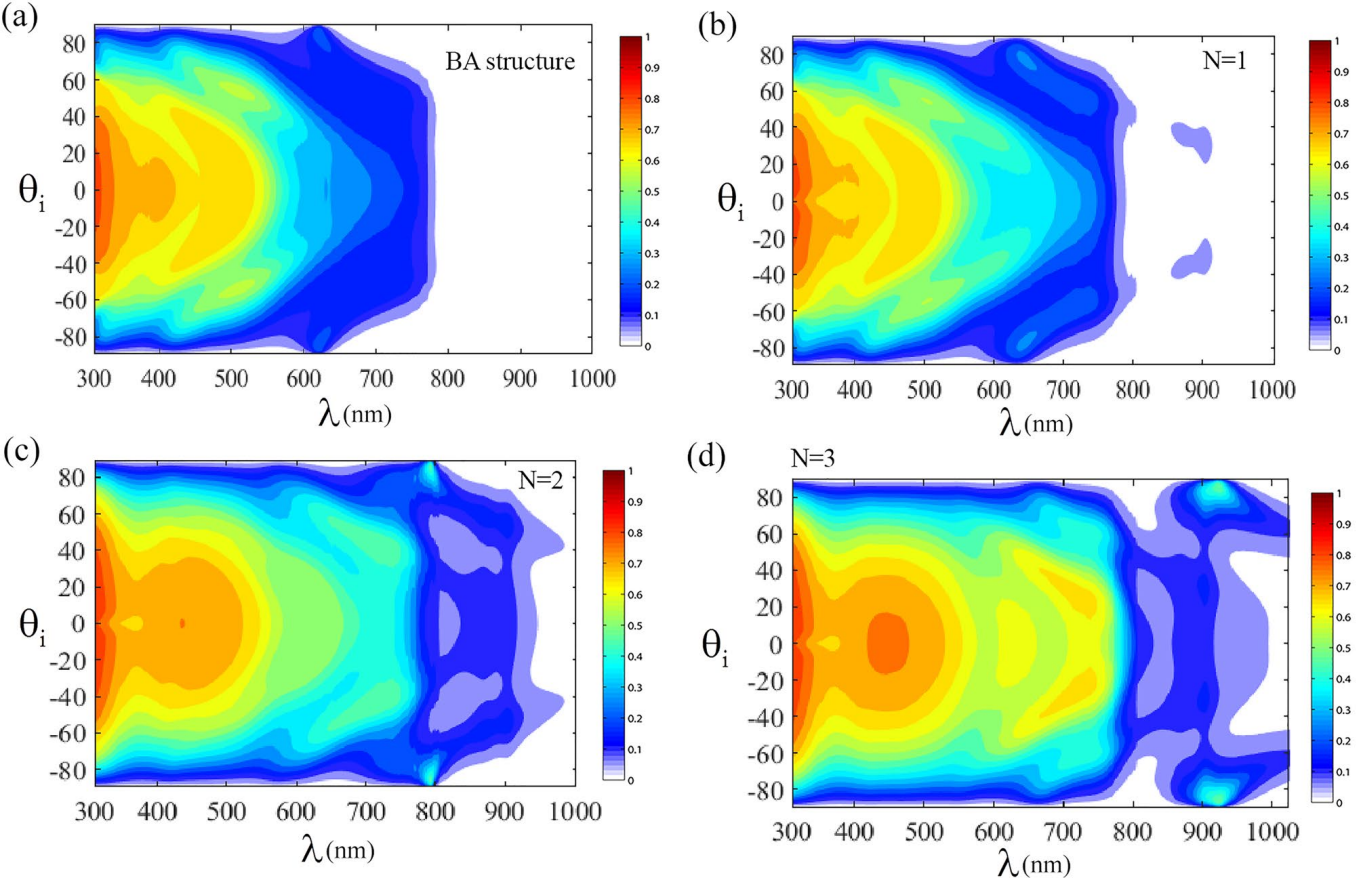
The absorbance coefficients as function of the wavelength of the incident light and the incident angle, (**a**) for *BA* structure, (**b**) *N* = 1, (**c**) *N* = 2 and (**d**) *N* = 3.

To further investigate the dependence of the absorbance coefficient on the incident light angle in the CH_3_NH_3_PbI_3_-based photonic crystal, the absorbance coefficient versus the incident light angle and wavelength of electromagnetic waves, for various number of periods is shown in Fig. 3. According to the figure, it is clear that for *BA* structure, for incident waves with longer wavelengths $$(\lambda > 800\begin{array}{*{20}c} \, {\rm nm} \\ \end{array} ),$$ the photonic crystal has no absorbance in this region. This phenomenon is because the CH_3_NH_3_PbI_3_ alone has very weak absorption in longer wavelengths^52,59^. Therefore, two different areas can be defined according to the wavelength of incident light: $$\lambda < 800\begin{array}{*{20}c} \, {\rm nm} \\ \end{array} ,$$ and $$\lambda > 800\begin{array}{*{20}c} \, {\rm nm} \\ \end{array} ,$$ in the first area, unlike the second area, the absorbance is non-zero. Thus, the absorbance versus the angle of the incident light and incident light wavelength has a gap. Moreover, for* N* = 1, a weak absorbance is observed around the incident wavelength of 900 nm, and with increasing number of periods, the absorbance occurs in most parts of the second region, due to the optical localization.

**Figure 4:**
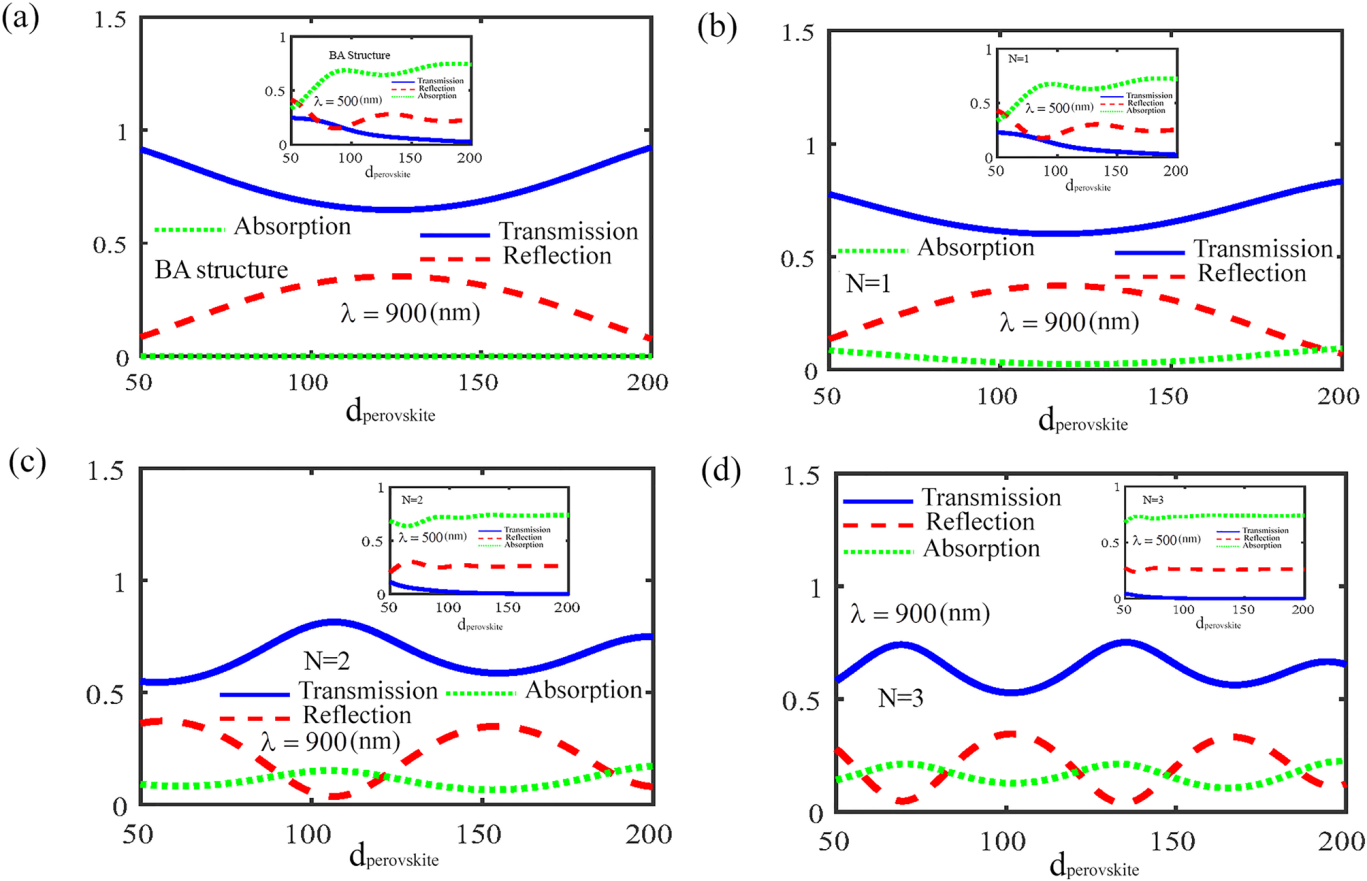
The dependence of the absorbance, transmittance and reflectance coefficients on the thickness of the CH_3_NH_3_PbI_3_ layer for the normal incident angle $\theta_{i} = 0^{o}$ (**a**) for *BA* structure, (**b**) *N* = 1, (**c**) *N* = 2 and (**d**) *N* = 3.

According to Figs. 2 and 3, the absorbance, reflectance and transmittance coefficients for different wavelengths of incident light in a CH_3_NH_3_PbI_3_ perovskite-based photonic crystal in the presence of monolayer MoS_2_ can be effectively controlled by the angle of the incident light and number of periods. In other words, absorbance coefficient depends on the number of periods and has an oscillatory behavior with respect to the angle of the incident light. This is because of the fact that the absorbance coefficient is an oscillating function of $\alpha_{j}$, which is determined by $\theta_{j}$. The dependence of the absorbance, transmittance and reflectance coefficients on the thickness of the CH_3_NH_3_PbI_3_ layer for the normal incident angle $\theta_{i} = 0^{o}$, is shown in Fig. 4. From Fig. 4, it is apparent that, for incident light in the visible region (for example, $$\lambda = 500\begin{array}{*{20}c} \, {\rm nm} \\ \end{array}$$), the transmittance coefficient decreases exponentially with increasing thickness of the CH_3_NH_3_PbI_3_ layer, due to the evanescent mode. Also, with increasing the number of periods, this decrease occurs faster, due to the strong light localization. Further, for visible incident light and for *BA* structure or *N* = 1, the absorbance and reflectance coefficients have an oscillatory behavior with respect to the thickness of the CH_3_NH_3_PbI_3_ layer. The reason for this behavior is the interface of CH_3_NH_3_PbI_3_ and the monolayer MoS_2_ layers. When the thickness of the CH_3_NH_3_PbI_3_ layer is large enough, transmittance tends to zero, due to the evanescent mode. As a result, with the increase in the number of periods of the photonic crystal, the oscillatory behavior of the absorbance and reflectance coefficients disappears and these quantities do not change significantly with increasing the thickness of the CH_3_NH_3_PbI_3_ layer. In addition, for infrared incident light (for example, $$\lambda = 900\begin{array}{*{20}c} \, {\rm nm} \\ \end{array}$$) and for *BA* structure the absorbance coefficient unlike the transmittance and reflectance coefficients, does not depend on the thickness of the CH_3_NH_3_PbI_3_ layer and has zero value, while for *N* = 1, absorbance can be observed at 900 nm. Moreover, with the increase in the number of periods of the photonic crystal, the absorbance coefficient increases and the oscillatory behavior of the absorbance, transmittance and reflectance coefficients becomes more obvious and prominent. Also, the oscillation period decreases with increasing *N*. Therefore, the amount of the absorbance, transmittance and reflectance coefficients in the CH_3_NH_3_PbI_3_ perovskite-based photonic crystal can be effectively tuned by controlling the thickness of the CH_3_NH_3_PbI_3_ layer.

**Figure 5:**
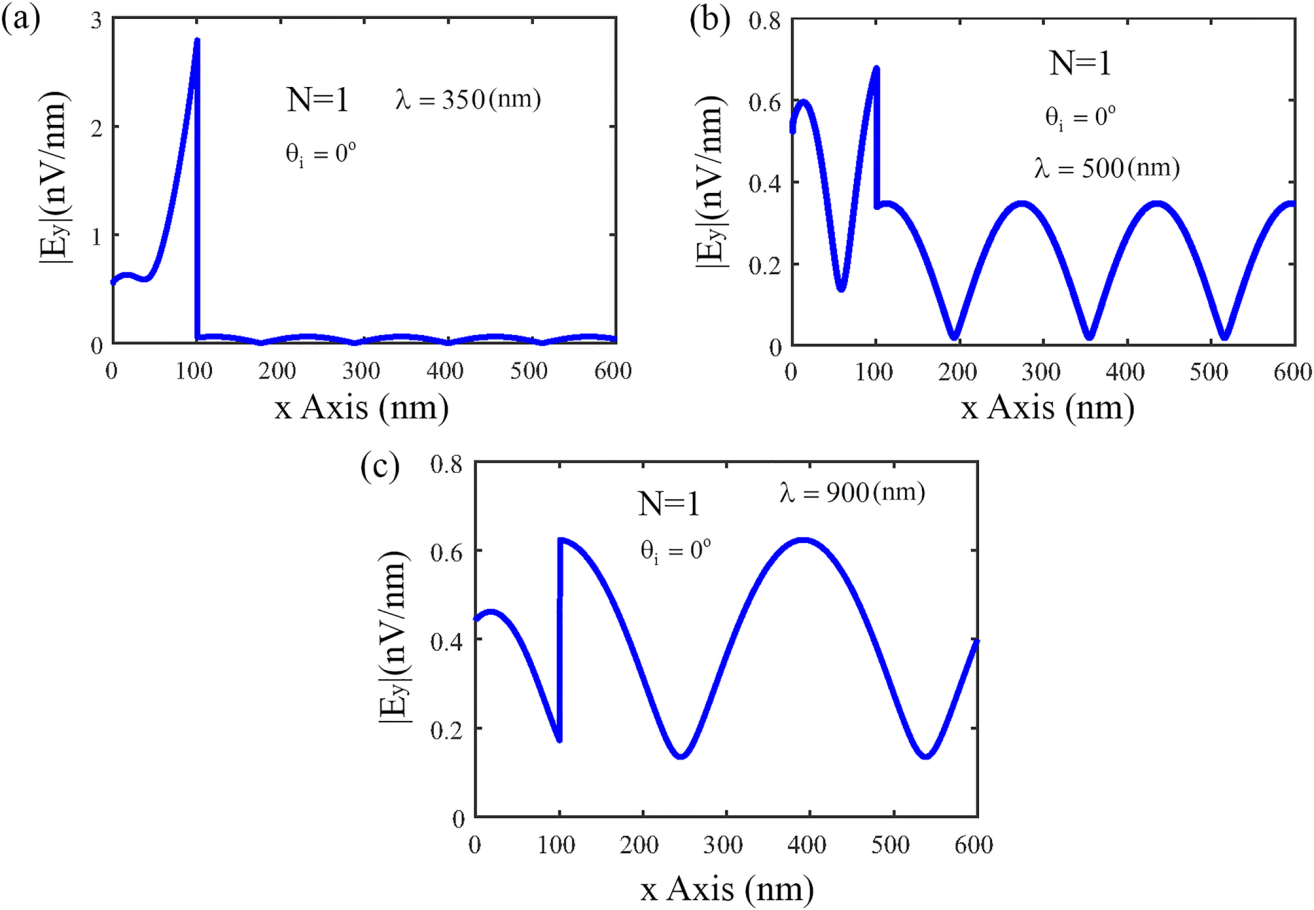
The distribution of electric field |*E*_*y*_| along *x*-direction for the normal incident angle $\theta_{i} = 0^{o}$. (**a**) for $$\lambda = 350\begin{array}{*{20}c} \, {\rm nm} \\ \end{array}$$, (**b**) $$\lambda = 500\begin{array}{*{20}c} \, {\rm nm} \\ \end{array} ,$$ and (**c**) $$\lambda = 900\begin{array}{*{20}c} \, {\rm nm} \\ \end{array}$$.

Figure 5 shows that the distribution of electric field |*E*_*y*_| along *x*-direction for the normal incident angle $\theta_{i} = 0^{o}$, and for different incident light wavelengths. According to the figure, it is clear that the distribution of the electric field in the *x-*axis in the SiO_2_ layer has an oscillatory behavior, and for incident light at small wavelengths, the amplitude of the oscillation is much smaller than the wavelengths of visible light and longer wavelengths. This result is completely consistent with the results obtained in Fig. 2a.

**Figure 6:**
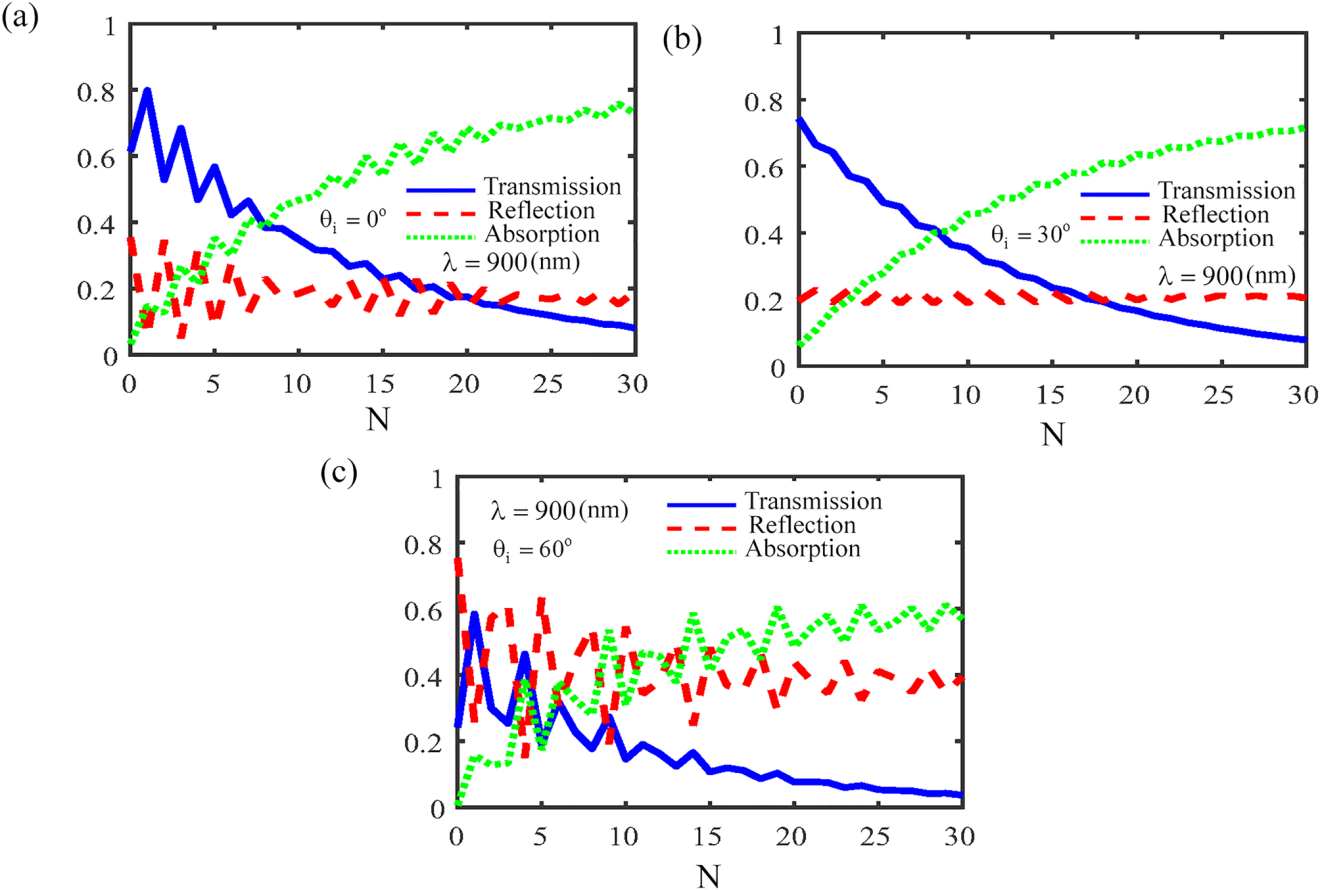
Absorbance, transmittance and reflectance coefficients as a function of the number of periods for different incident light angles for $$\lambda = 900\begin{array}{*{20}c} \, {\rm nm} \\ \end{array}$$. (**a**) for $\theta_{i} = 0^{o}$, (**b**) $\theta_{i} = 30^{o}$ and (**c**) $\theta_{i} = 60^{o}$.

Absorbance, transmittance and reflectance coefficients as a function of the number of periods for different incident light angles and for $$\lambda = 900\begin{array}{*{20}c} \, {\rm nm} \\ \end{array}$$ are shown in Fig. 6. As can be seen, with the increase in the number of periods, the transmittance coefficient decreases and for large values of the number of periods it reaches a very small value, due to strong light localization. Also, by increasing the number of periods of the photonic crystal, the reflectance coefficient first shows an oscillatory behavior, and this oscillatory behavior is highly dependent on the incident light angle. While further increasing the number of periods, the value of reflectance becomes constant. Therefore, by increasing the number of periods, the absorbance of the photonic crystal increases and finally reaches its maximum constant value, and this maximum value decreases with the increase of the incident light angle. Thus, by controlling the number of periods, it is possible to obtain a photonic crystal based on CH_3_NH_3_PbI_3_, in which the absorbance, transmittance and reflectance coefficients have their optimal values. In other words, localization effect of light can enhance the absorption^61–63^. Thus, one-dimensional CH_3_NH_3_PbI_3_ perovskite-based photonic crystal in the presence of the monolayer MoS_2_ can be used to significantly enhance the absorption in particular areas, due to the strong interference effect in the interface of CH_3_NH_3_PbI_3_ and the monolayer MoS_2_ layers. These results indicate that, the CH_3_NH_3_PbI_3_ perovskite-based photonic crystal in the presence of monolayer MoS_2_ film could be a good candidate for further enhancement the light absorbance and transmittance in optoelectronic and optical devices. For example solar cells based on CH_3_NH_3_PbI_3_ and infrared sensor system^64^.

## Conclusion

In conclusion, we studied theoretically the electromagnetic wave propagating in a CH_3_NH_3_PbI_3_ perovskite-based photonic crystal. We showed that, for *BA* structure the absorbance coefficient of the structure is zero for $$\lambda > 800\begin{array}{*{20}c} \, {\rm nm} \\ \end{array} ,$$ while for *N* = 1 the photonic crystal has a weak absorbance around 900 nm, Also, due to the optical localization in CH_3_NH_3_PbI_3_-based photonic crystal in the presence of the monolayer MoS_2_ film_,_ this structure enhances the light absorption at longer wavelengths. This phenomenon will be more evident with the increase in the number of periods. Furthermore, for *BA* structure the absorbance coefficient of the structure versus the angle of the incident light and incident light wavelength has a gap. With increasing number of periods, absorbance occurs for incident wavelengths greater than 800 nm. Thus, the angle of the incident light, thickness of the CH_3_NH_3_PbI_3_ layer and number of periods are key parameters to controlling the absorbance, reflectance and transmittance coefficients in a CH_3_NH_3_PbI_3_ perovskite-based photonic crystal in the presence of monolayer MoS_2_ film. Moreover, the absorbance, transmittance and reflectance coefficients have an oscillatory behavior with respect to the thickness of the CH_3_NH_3_PbI_3_ layer, and with the increase in the number of periods of the photonic crystal, the absorbance increases and the oscillatory behavior of the absorbance, transmittance and reflectance coefficients becomes more obvious and prominent. In addition, by increasing the number of periods, the absorbance of the photonic crystal increases and finally reaches its maximum constant value for *N* = 30, and this maximum value decreases with the increase of the incident light angle. So that the maximum value of the absorbance coefficient for incident light angle of $\theta_{i} = 0$°, $\theta_{i} = 30$° and $\theta_{i} = 60$° will be 80%, 70% and 60%, respectively. Thus, by controlling the number of periods, it is possible to obtain a photonic crystal based on CH_3_NH_3_PbI_3_, in which the absorbance, transmittance and reflectance coefficients have their optimal values. The present investigation showed that, the CH_3_NH_3_PbI_3_ perovskite-based photonic crystal can be useful for efficient photoabsorber devices, such as solar cells and infrared sensor system.

## Data Availability

The datasets used and/or analysed during the current study are available from the corresponding author on reasonable request.
